# Beyond Observable Behavior: A Narrative Review of Integrated Clinical, Biological, and Functional Assessment in Children With Autism Spectrum Disorder

**DOI:** 10.7759/cureus.118440

**Published:** 2026-10-23

**Authors:** Florencia Sanabria

**Affiliations:** 1 Neurodevelopment, Instituto HELMAR, Mar del Plata, ARG

**Keywords:** autism spectrum disorder (asd), clinical assessment tools, #clinical decision-making, differential diagnosis, functional assessment, medical comorbidities, neurodevelopmental disorders (nd), pediatric neurodevelopment, smart-nb, therapeutic goals

## Abstract

Assessment of children with autism spectrum disorder must characterize everyday functioning, co-occurring conditions, and individual support needs alongside diagnostic classification. This narrative review examines the evidence and clinical rationale for integrated clinical, biological, and functional assessment in pediatric autism and presents SMART-NB as a proposed framework for organizing these dimensions. The review combines selected clinical guidelines and research publications with conceptual analysis and a targeted literature update, distinguishing established assessment principles from the author-developed framework. Three findings emerge. First, standardized observational instruments contribute to diagnosis but require interpretation alongside developmental history, caregiver information, differential diagnosis, and clinical judgment. Second, co-occurring sleep disturbances, gastrointestinal symptoms, nutritional problems, sensory impairments, and pain can influence behavior and participation and warrant clinically directed evaluation; their association with autism does not establish causation. Third, symptom severity does not fully describe adaptive functioning, supporting assessment across functional domains and everyday contexts. SMART-NB proposes connecting clinical, medical and biological, and demand-related profiles with seven functional domains, individualized goals, and longitudinal reassessment. The demand dimension examines available capacity, physiological and emotional burden, environmental requirements, and supports. Its proposed contribution is to document how assessment findings inform care priorities. Existing multidimensional approaches already address overlapping aspects of this task; the incremental value of this particular organization remains untested. The reviewed evidence supports multidimensional assessment but does not establish the validity, reliability, or clinical utility of SMART-NB. This review is not systematic, and extension beyond autism remains a hypothesis requiring condition-specific evaluation. Development should proceed from operational specification, independent content assessment, and feasibility studies to the evaluation of measurement properties and comparative clinical outcomes.

## Introduction and background

A diagnosis of autism spectrum disorder identifies a clinically meaningful developmental pattern, but it does not by itself explain a child's communication resources, physical discomfort, daily independence, or priorities for intervention. Diagnostic assessment and functional characterization answer related questions. Clinical standards therefore combine developmental history, direct observation, information from caregivers and relevant settings, differential diagnosis, and assessment of accompanying needs [1,2,3]. Contemporary reviews also describe substantial heterogeneity within autism, including differences in language, intellectual development, associated conditions, and support requirements [4,5].

Standardized instruments can strengthen assessment when used for their intended purpose. NICE explicitly advises against diagnosing autism from an autism-specific tool alone [6]. The relevant clinical question is how findings from instruments, history, examination, and everyday participation should be integrated. This review does not assume that contemporary guidelines advocate isolated behavioral classification or that multidisciplinary assessment is a new principle.

Medical and physiological assessment contributes to this formulation. Sleep disruption, gastrointestinal difficulties, restricted dietary intake, sensory impairment, epilepsy, pain, and treatment effects may coexist with autism and influence functioning. These possibilities require individualized evaluation rather than the assumption that every behavioral manifestation has a separate medical explanation [7,8]. Psychological, relational, educational, and environmental factors also require explicit consideration.

SMART-NB is the author's proposed organization of this information into an assessment-to-priority process. Its documentary antecedents are the SMART-NB White Paper v1.0 and the author's books *No todo es autismo: 100 razones para mirar más allá* and *Niños bajo presión: El estrés infantil disfrazado de trastorno* [9,10,11]. The White Paper explicitly identifies itself as an institutional document that has not undergone peer review and remains at the pre-validation stage. The books are cited as conceptual antecedents, not as peer-reviewed empirical validation. External literature supports the assessment principles discussed here; it does not validate the SMART-NB architecture, scores, or clinical effects.

The primary objective is to synthesize the rationale for integrated assessment in children with autism and clarify how SMART-NB proposes linking findings with individualized functional priorities. Secondary objectives are to compare the proposal with existing frameworks, define its components sufficiently for independent scrutiny, and distinguish its conceptual claims from questions requiring empirical evaluation. Autism provides the main evidence base. Broader neurodevelopmental and psychiatric applications are considered only as proposed extensions.

## Review

Methodological approach

This article is a narrative review with conceptual analysis, not a systematic review, scoping review, or meta-analysis. The source corpus combines the bibliography of the earlier manuscript with a targeted revision update performed on October 17, 2026. The update searched PubMed through the NCBI interface and used journal websites, institutional repositories, and official sources from the WHO, NICE, the Canadian Paediatric Society, ZERO TO THREE, and instrument publishers. Crossref and DataCite were used for bibliographic verification; registry metadata were not treated as evidence of study quality or framework validation.

Searches addressed autism assessment and differential diagnosis; observational-tool specificity and task demands; medical conditions and functioning; adaptive trajectories; integrated developmental frameworks; individualized goal setting; case-formulation reliability; and measurement development. Representative PubMed combinations were “autism AND diagnostic assessment,” “autism AND ADOS AND specificity,” “autism AND adaptive functioning AND longitudinal,” and “case formulation AND reliability.” The update covered publications dated from January 1, 1900, through October 17, 2026, without a narrower starting-date restriction. English-language literature predominated; Spanish-language author publications were considered only for conceptual provenance.

Selection was purposive and question-led. Sources were retained when they informed pediatric autism assessment, differential diagnosis, medical or functional characterization, an explicitly compared framework, or the development and evaluation of measurement and clinical processes. Clinical guidance and evidence syntheses were prioritized for established principles; original studies were used for specific empirical observations. Methodological work outside pediatric autism was retained for its methodological relevance, with transfer limitations stated. Adult-only clinical findings, duplicate presentations of the same result, and publications without a direct connection to the review questions were not used as independent support for pediatric or SMART-NB claims. Apparent contradictions were retained when they limited interpretation rather than removed to create consensus.

The analysis organized information by clinical question, population, design, principal finding, and limitation. It separated empirical findings, guideline recommendations, conceptual comparisons, and author-proposed specifications. There was no independent duplicate screening, formal study-level risk-of-bias assessment, certainty grading, or statistical pooling. The earlier manuscript did not report historical search dates or exclusion counts; these have not been reconstructed for this revision. The final bibliography contains 74 sources; this is a citation count, not a claim that every search result was screened or that the evidence base was exhaustively captured. Bibliographic records and abstracts were used for verification and source identification; they cannot substitute for full-text critical appraisal. These features limit reproducibility and certainty.

SANRA informed revision of the review's rationale, aims, search description, referencing, scientific reasoning, and presentation of relevant evidence [12]. No SANRA score or certification of review quality is claimed. The framework was examined as an author-developed conceptual proposal; no new patient dataset, validation sample, clinical experiment, or diagnostic-accuracy analysis was generated.

Findings supporting integrated assessment

The reviewed literature supports three complementary conclusions. Diagnostic observations require developmental and contextual interpretation; medical and psychological conditions can add clinically important needs; and diagnostic severity does not fully describe adaptive functioning. These conclusions support coordinated assessment and individualized planning. They do not establish a single biological explanation for autism or demonstrate that one organizational framework is superior [1-3,13].

The distinction between diagnosis and functioning is supported by empirical work. Kanne and colleagues examined adaptive behavior in 1,089 verbal young people with autism and reported substantial adaptive difficulties, with negligible associations between autism symptomatology and adaptive behavior in that sample [13]. Longitudinal research has identified distinct trajectories of symptom severity and adaptive functioning and differences across adaptive domains [14,15]. These findings concern particular populations and measures. They justify assessing adaptive needs directly rather than assuming a universal relationship between symptoms and functioning.

The literature also supports careful use of standardized tools. A recent scoping review of early developmental assessment identified evidence-based instruments across multiple domains and emphasized selection according to purpose, population, accuracy, resources, and accessibility [16]. An international outcome-measure consensus for communication, attention, learning, and motor disorders similarly demonstrates that transdiagnostic outcome monitoring already has established methodological precedents [17]. Neither publication evaluates SMART-NB.

Observational instruments and clinical interpretation

ADOS and its revised algorithms were developed to provide standardized opportunities to observe autism-related social and communicative behavior [18,19]. Combining observational findings with developmental interview information can contribute complementary evidence [20]. Independent diagnostic-accuracy research shows that performance varies across modules and comparison populations [21]. These observations support skilled use of instruments within a complete assessment.

Greene and colleagues examined 214 children and adolescents aged five to 16 years assessed with ADOS-2 Module 3 in a clinical referral setting. Anxiety during assessment and trauma-related diagnoses were relevant to false-positive classifications in that sample [22]. This does not establish a universal false-positive rate across ADOS-2 modules, referral pathways, or populations. Similarly, the study by Klein-Tasman and colleagues showed that language and task demands, including module choice, can influence observed classification [23]. It does not show that autism is merely an effect of task demands.

An observational classification, a clinical diagnosis, and an etiological explanation should therefore be documented as different outputs. Clinical disagreement should prompt review of developmental history, test conditions, differential diagnoses, and information across settings. The limitations of an established instrument do not constitute evidence for the accuracy or superiority of SMART-NB [6].

Differential diagnosis and overlapping presentations

Clinical reasoning must allow both alternative explanations and co-occurrence. Intellectual disability, language disorders, ADHD, anxiety, and other presentations can share features with autism without being interchangeable diagnoses [3,24,25]. Population-based evidence also documents accompanying psychiatric and developmental conditions in autistic children and adolescents [26]. A single behavior cannot determine which diagnosis or combination of diagnoses is supported.

Assessment of children with limited spoken language requires particular care. Communication modality, receptive abilities, nonverbal cognition, intentional communication, play, and context may be poorly represented by a brief encounter or a measure with floor effects [27,28]. Limited speech should not be equated with uniformly limited understanding. Equally, a skill observed once should not be assumed to be consistently available across settings.

Diagnostic overshadowing is a relevant possibility, but evidence varies by population and study design [29]. The proposed formulation should record which diagnoses are established, which remain hypotheses, what evidence supports them, and what further information could change the interpretation. It should not attribute pain, anxiety, or regression automatically to autism, nor infer the absence of autism from improvement after treatment of a co-occurring condition.

Medical and biological characterization

The term organic profile is retained from the author's framework and defined here as the medical and physiological component of case formulation. It includes established etiologies, co-occurring conditions, possible contributors to current difficulties, and treatment effects. It is not a binary division between organic and psychological illness and does not imply that every behavioral difficulty has an identifiable medical cause [7,8].

History and examination should guide further investigation. Relevant questions include the timing of changes, regression, sleep, pain, feeding, growth, gastrointestinal symptoms, sensory access, seizures, medication exposure, and family history. NICE advises against routine indiscriminate medical investigation during autism diagnostic assessment, while identifying circumstances in which specific investigations are appropriate [6]. Hearing or visual impairment may complicate observation and communication and warrants consideration on its own clinical merits [30].

Sleep problems and epilepsy can add distinct needs, but their evaluation depends on clinical presentation [31,32]. Genetic evaluation should follow the child's phenotype and applicable professional guidance. Evidence supporting exome or genome sequencing in congenital anomalies, developmental delay, or intellectual disability must not be translated into a universal recommendation for all children referred for autism assessment [33-35]. An etiological diagnosis and a functional description remain complementary outputs.

Gastrointestinal and feeding difficulties also merit clinically directed evaluation. Gastrointestinal symptoms may be associated with behavioral and communication difficulties, with uncertain causal direction [36,37]. Feeding and nutrition syntheses identify increased feeding problems and group-level nutritional differences; these do not diagnose a deficiency in an individual child or establish a universal dietary treatment for autism [38,39]. Pain may be expressed through changes in activity, communication, or behavior and can be difficult to recognize when expressive communication is limited [40]. Medication benefits and adverse effects should be considered when changes in alertness, sleep, appetite, regulation, or participation coincide with treatment changes [41].

Relationship to existing integrated frameworks

Integrated assessment predates SMART-NB. ESSENCE directs attention to overlapping early developmental difficulties and the need for input across disciplines rather than restricting assessment to the initial diagnostic label [42]. DC:0-5 provides a developmentally sensitive multiaxial diagnostic approach for infancy and early childhood that considers relationships, physical health, psychosocial context, and developmental competence [43]. Neither should be reduced to a single symptom checklist.

The ICF describes functioning through interactions among health conditions, body functions and structures, activities, participation, and environmental factors. The 2007 ICF-CY is an important historical pediatric source [44]. WHO now states that ICF-CY has been merged into the ICF and is no longer separately supported [45]. Accordingly, the present review acknowledges pediatric development through ICF-CY while using current ICF terminology. Autism-specific ICF Core Sets and subsequent applications provide direct precedents for multidimensional functional characterization [46-48].

Ei SMART is a separate integrated early-intervention framework concerning interrelated sensory, motor, attention and regulation, and relational development with collaboration between families and professionals [49,50]. Evidence concerning its principles or implementation cannot be transferred to SMART-NB solely because the names overlap. Likewise, SMART goal-setting criteria describe the construction of objectives rather than a diagnostic or biological model [51]. The present comparison establishes conceptual relationships; it does not assert a documented historical derivation of SMART-NB from either use of SMART.

Table 1 compares the purposes and boundaries of established approaches with the contribution proposed here.

**Table 1:** Comparison with existing frameworks. Source: [17,42,43,45,49]. The comparison is conceptual; no comparative effectiveness study was undertaken. ESSENCE, Early Symptomatic Syndromes Eliciting Neurodevelopmental Clinical Examinations; ICF, International Classification of Functioning, Disability and Health; ICF-CY, International Classification of Functioning, Disability and Health for Children and Youth; WHO, World Health Organization

Approach	Existing contribution	Relationship proposed for SMART-NB	Boundary of the comparison
ESSENCE	Recognizes overlapping developmental difficulties and multidisciplinary needs	Organize identified needs into explicit current priorities and reassessment	Does not establish a novel transdiagnostic principle or demonstrate added benefit
DC:0-5	Developmentally sensitive multiaxial formulation in early childhood	Carry contextual and health information into a functional priority record	Different age remit; no equivalence with its diagnostic system
ICF and pediatric antecedent ICF-CY	Describes functioning, participation, and environmental influences	Link functional findings with diagnostic hypotheses and selected actions	Seven SMART-NB domains are not validated ICF codes or an official WHO instrument
Ei SMART	Integrates developmental systems and family-professional collaboration in early intervention	Compare how integrated information supports planning	Separate framework; shared terminology does not transfer validation
International outcome consensus	Defines transdiagnostic outcomes and recommended measures	Inform selection of outcomes for future SMART-NB studies	Existing consensus does not validate the proposed framework.
ESSENCE	Recognizes overlapping developmental difficulties and multidisciplinary needs	Organize identified needs into explicit current priorities and reassessment	Does not establish a novel transdiagnostic principle or demonstrate added benefit
DC:0-5	Developmentally sensitive multiaxial formulation in early childhood	Carry contextual and health information into a functional priority record	Different age remit; no equivalence with its diagnostic system
ICF and pediatric antecedent ICF-CY	Describes functioning, participation, and environmental influences	Link functional findings with diagnostic hypotheses and selected actions	Seven SMART-NB domains are not validated ICF codes or an official WHO instrument
Ei SMART	Integrates developmental systems and family-professional collaboration in early intervention	Compare how integrated information supports planning	Separate framework; shared terminology does not transfer validation
International outcome consensus	Defines transdiagnostic outcomes and recommended measures	Inform selection of outcomes for future SMART-NB studies	Existing consensus does not validate the proposed framework.

The resulting research question is narrower than whether integrated care is useful: does documenting a specific chain from findings to formulation, priorities, goals, and reassessment improve decisions or outcomes beyond existing practice? The present literature cannot answer that question. The proposed contribution is an explicit organizational process; originality of an arrangement is not evidence of clinical advantage.

Definition and scope of SMART-NB

SMART-NB is proposed as a clinical organization and longitudinal follow-up framework. NB refers to the author's Spanish term neurodesarrollo biológico, emphasizing the medical and physiological context of development. It does not denote a validated biological subtype or biomarker classification. The White Paper documents the longitudinal architecture, functional domains, and provisional Global Functional Index (GFI) [9]. The clinical, organic, and demand formulations described here organize complementary information within that architecture.

The intended starting population for evaluation is children referred for suspected or established autism who require an individualized account of functioning and care priorities. Information may be contributed by families, educators, and professionals. Medical diagnosis and decisions about investigations or medication remain within the responsibilities of appropriately qualified clinicians. The model is not a diagnostic test, a substitute for professional judgment, or a rule requiring every child to undergo the same assessment battery.

Clinical profiles and evidence boundaries

The clinical profile records developmental history, confirmed diagnoses, diagnostic hypotheses, onset, change from baseline, strengths, and co-occurring needs (Table 2). Ten non-exclusive working profiles are retained from the author's specification [9]. Their arrangement belongs to SMART-NB; it is not a new Diagnostic and Statistical Manual of Mental Disorders, Fifth Edition, Text Revision (DSM-5-TR) classification. Anxiety, depression, psychotic disorders, and oppositional defiant disorder should not all be described as neurodevelopmental disorders under DSM-5-TR [52].

**Table 2:** Clinical profiles and limits of extrapolation. Sources: [3,24-26,52-58]. Evidence supports the stated assessment principles; no row establishes condition-specific validation of SMART-NB. ADHD, attention-deficit/hyperactivity disorder

Working profile	Relevant established assessment principle	Evidence boundary for SMART-NB
ADHD	Developmental and psychiatric history, impairment across settings, and co-occurring needs	Condition-specific assessment is supported; the SMART-NB organization is untested
Communication and language	Assess receptive, expressive, pragmatic, and accessible communication	Overlap with autism requires differential interpretation
Autism spectrum disorder	Integrate developmental history, observation, differential diagnosis, and functioning	Main population addressed by this review
Motor disorders	Characterize mobility, coordination, praxis, and effects on participation	Motor difficulties in autism do not establish one common mechanism
Intellectual disability	Evaluate intellectual and adaptive functioning in developmental context	Limited speech alone is insufficient for this classification
Specific learning disorder	Assess educational opportunity, instruction, academic skills, and alternatives	School-age assessment principles do not validate a seven-domain index
Anxiety disorders	Examine fears, avoidance, physiological arousal, and functional interference	Anxiety may coexist with autism or affect assessment performance
Depressive disorders	Assess persistent mood change, safety, functioning, and contextual factors	Evidence for depression assessment is not evidence for this framework
Psychotic symptoms or disorders	Specialist assessment of developmental context, mental state, medical factors, and safety	Age-sensitive differential assessment; no routine psychosis screen implied for every young child
Oppositional defiant disorder	Evaluate persistent patterns, context, communication, and co-occurrence	Refusal or distress in a single setting does not establish the diagnosis

Evidence beyond autism includes clinical guidance for ADHD, learning disorders, depression, psychosis, and disruptive behavior, and transdiagnostic outcome research. These sources support attention to context, co-occurrence, and functioning within their respective populations. They do not establish that SMART-NB classifications, domain ratings, or priorities have equivalent validity across diagnoses. Each proposed extension requires appropriate age, language, cultural, and condition-specific evaluation.

Medical and contextual profiles

The medical and biological record should distinguish a confirmed condition from a suspected contributor and from information not yet assessed. Each entry should state its source, date, relevant findings, and implications for the child's current needs. An abnormal test result is not automatically an explanation of behavior. A medical cause should not be inferred solely from a domain score or demand label.

Context is documented across all profiles: family routines and resources, educational opportunity, language and culture, relationships, trauma or stress, access barriers, accommodations, and the child's preferences. The child and family are sources of knowledge and partners in prioritization, rather than causes presumed to account for a difficulty. Family-centered practice and developmental-behavioral pediatrics provide established precedents for this approach [59,60].

Capacity burden and contextual demand

The demand dimension asks what the child can currently do, what an activity requires, what supports are available, and which physiological or emotional burdens may constrain performance. Capacity and performance are distinguished in ICF terminology [45]. Poor performance alone cannot show whether a difficulty reflects limited capacity, distress, task conditions, inaccessible communication, or several interacting factors.

The earlier labels low demand and high demand are potentially misleading. In this review, the corresponding working descriptions are capacity-constrained presentation and burden- or context-constrained presentation. These are proposed formulations, not diagnostic categories. A capacity-constrained presentation means that a relevant skill remains unavailable or limited across appropriately supported observations. It does not mean that the environment demands little or that support needs are low. A burden- or context-constrained presentation means that performance varies in relation to identifiable burdens or requirements; prior performance alone does not prove that capacity is stable or sufficient.

Mixed and uncertain formulations are permitted. A child with a communication limitation can simultaneously experience pain, anxiety, fatigue, or inaccessible classroom demands. Documentation should retain both dimensions rather than force a binary assignment. The proposed record specifies the activity, evidence of ability, task demands, supports, changes from baseline, and competing explanations. No quantitative cutoff, causal mechanism, or classification accuracy is claimed. If available information cannot discriminate explanations, the formulation remains uncertain and the next observation or evaluation is stated.

Seven functional domains

Seven domains organize descriptive information without requiring seven separate diagnoses. Table 3 gives working definitions and example observations. These definitions clarify the proposal for testing; they have not undergone independent content validation. Measures should be selected according to the question and relevant psychometric evidence, and should not be replaced by an impressionistic domain label.

**Table 3:** Working definitions of the functional domains. Source of the proposed domain organization: [9]. Examples are conceptual documentation prompts, not test items or validated scoring criteria.

Code and domain	Working definition	Example information to document
COM Communication and language	Understanding and conveying meaningful information through available modalities	Comprehension, requests, expression of discomfort, augmentative communication, partner and setting
LEARN Learning and problem solving	Acquiring, retaining, and applying skills in relevant contexts	Demonstration, retention, transfer, instruction received, opportunity to learn
EXEC Attention and executive functions	Initiating, sustaining, organizing, adapting, and completing goal-directed activity	Task length, transitions, prompts, planning, environmental demands
ADP Adaptive autonomy	Performing daily routines and assuming developmentally appropriate responsibilities with support	Self-care, safety, daily organization, type and amount of assistance
SOC Social participation	Accessing relationships and participating in family, school, peer, and community activities	Engagement, communication access, preferences, barriers, belonging
MOT Motor skills and praxis	Using movement, coordination, and motor planning in everyday activities	Mobility, manipulation, fatigue, accessibility, adaptive equipment
REG Regulation and self-management	Using co-regulation, self-regulation, and accessible help-seeking to manage internal states and activity	Recovery from distress, supports, triggers, communication of needs

Each domain record should identify the task, setting, informant, communication mode, assistance, consistency, and date. Skills not adequately observed remain unknown. Conflicting caregiver, school, and session observations should be retained and investigated rather than mechanically averaged. Chronological age and domain-specific functioning are recorded separately; the profile does not assign a global mental age or justify age-inappropriate goals.

Provisional ratings and the Global Functional Index

The White Paper proposes ordinal domain anchors from zero to four: function unavailable or highly dependent despite support; emerging or inconsistent with intensive support; partial with moderate support in limited contexts; functional in usual settings with minimal or occasional support; and consistent, sufficiently generalized function for current demands [9]. These anchors combine several constructs, including capacity, assistance, consistency, and generalization. Their clarity, dimensional structure, and reproducibility require testing before they can be interpreted as measurement.

The documented pilot formula is GFI = 25 × the mean of available domain ratings, giving a theoretical range from zero to 100. It is reported to make the source specification transparent, not endorsed as an established score. Arithmetic aggregation assumes equal weighting and comparability of ordinal categories, neither of which has been demonstrated. Missing domains are not scored as zero. Different numbers or sets of observed domains can change the meaning of a total; longitudinal comparisons must therefore identify the same observed domains and conditions, or avoid interpreting change in the aggregate.

A heptagonal display can communicate a profile, but polygon area depends on axis order and geometry and is not an outcome measure. Neither a visually larger polygon nor a higher provisional GFI establishes meaningful improvement, diagnostic severity, treatment efficacy, or eligibility for services. Domain-level observations and established measures retain interpretive priority. The present review reports no numerical SMART-NB results.

From formulation to therapeutic priorities

The proposed clinical output is a dated record connecting a finding to its interpretation, current priority, agreed goal, planned action, responsible professional, and review condition. Immediate safety and medical needs take precedence when present. Access to communication, comfort, meaningful participation, and family feasibility guide the remaining priorities. Medical evaluation, educational accommodations, and appropriate intervention may proceed in parallel; completing every investigation is not a prerequisite for support [1,61].

For example, an illustrative child may have an established autism diagnosis, limited means to request help, recent sleep disruption, and distress during lengthy classroom activities. The formulation can retain autism, identify sleep as a separate clinical concern, and prioritize accessible requesting and a change in task conditions. This is a hypothetical illustration, not an observed case or evidence that the framework improves outcomes.

An individualized goal specifies baseline performance, activity, context, communication modality, allowed assistance, an observable criterion, and a review interval. A hypothetical example is requesting help through an established communication system in four of five naturally occurring opportunities during a classroom routine over three observed days, with no more than one agreed prompt, reviewed after eight weeks. The numbers illustrate goal construction and are not validated SMART-NB thresholds. SMART goal-setting research and Goal Attainment Scaling provide related precedents, with methodological limitations of their own [51,62,63].

Relationship to established functional measures

Vineland-3 and ABAS-3 assess adaptive behavior; their manuals or official technical sources should support descriptions of their constructs [64,65]. A critical appraisal of adaptive social communication measurement can inform limitations, but does not replace those original instrument sources [66]. PEDI-CAT addresses functional performance in daily activities, mobility, social and cognitive functioning, and responsibility; its development study evaluated accuracy and precision relative to its item banks [67]. GAS addresses attainment of individualized objectives [62].

These instruments are not interchangeable and none is a universal reference standard for the whole SMART-NB proposal. The framework may organize findings from appropriately selected measures while retaining their original scoring and interpretation. Correlations with one established measure would not establish comprehensive validity, and using validated instruments within an assessment does not validate the framework that assembles them.

Longitudinal reassessment and case formulation reliability

Reassessment should examine whether goals remain relevant, skills generalize, assistance changes, and new health or contextual issues emerge. Domain-specific trajectories and changes in symptoms or adaptive functioning justify revisiting priorities rather than equating diagnostic stability with stable needs [14,15,68]. Review intervals should reflect the goal and clinical circumstances; no optimal SMART-NB interval has been established.

A structured formulation is still a clinical judgment that may vary across evaluators. A systematic review of case-formulation reliability found variable agreement and methodological weaknesses, with some evidence that training and experience matter [69]. This is relevant methodological background, not pediatric autism validation. SMART-NB should therefore preserve source information and explicit reasoning and undergo direct inter-rater assessment. Agreement about a formulation, accuracy of its causal interpretation, and benefit to the child are separate questions.

Independent development and evaluation

The clinical process and its numerical outputs require different evaluation pathways. Guidance for complex interventions is relevant to the organization of assessment and decision-making [70]. Measurement-development guidance supports defining constructs and intended uses before studying reliability, validity, measurement error, and responsiveness [71-73]. COSMIN terminology originated largely in health-outcome measurement; its application should match the type of instrument rather than be treated as blanket certification (Table 4).

**Table 4:** Proposed evaluation sequence. Sources: [70-74]. The table presents a proposed research sequence; no listed stage has been completed in the present review.

Stage	Principal question	Suitable evidence or design	What the stage cannot establish alone
Operational specification	Can users identify the population, profiles, domains, and rules?	Versioned manual, worked examples, rules for overlap and missing data	Clinical efficacy
Independent content assessment	Are elements relevant, sufficiently complete, and understandable?	Expert and family input; structured consensus such as Delphi	Diagnostic accuracy or improved outcomes
Feasibility and acceptability	Can the process be used without disproportionate burden?	Prospective pilot assessing time, training, completion, accessibility, and acceptability	Psychometric validity or comparative benefit
Reliability and measurement error	Do defined ratings agree under appropriate conditions?	Trained independent raters, predefined statistics, repeated observations where stability is plausible	Accuracy of causal explanations
Construct validity and responsiveness	Do ratings behave as specified and detect meaningful change?	Prespecified hypotheses, suitable measures, longitudinal anchors, analysis of missingness	Superiority over usual care
Comparative clinical utility	Does the framework add benefit to existing assessment?	Prospective controlled implementation comparing decisions, function, family outcomes, costs, and harms	Transfer to unstudied settings or diagnoses

The seven-domain profile should not automatically be modeled as a single reflective scale. If domains form a composite rather than reflect one latent trait, internal consistency alone is not an adequate validation strategy. Domain ratings, the provisional GFI, and the organizational process should be evaluated separately. Studies should prespecify outcomes and consider assessment time, unnecessary testing, treatment burden, access, family priorities, adverse consequences, and conflicts of interest. Independent teams and external settings are necessary to test transportability.

Limitations

The narrative selection is vulnerable to publication, language, accessibility, and author-selection biases. A targeted update improves transparency but does not retrospectively convert the review into a systematic search. Without duplicate screening, formal risk-of-bias assessment, or certainty grading, the synthesis cannot establish completeness or pooled effectiveness. Source metadata verification confirms identity and citation details; it does not replace substantive appraisal of full texts.

Most empirical discussion concerns autism, and samples vary in age, language, intellectual functioning, referral setting, and instruments. Findings from a specific ADOS module or clinical subgroup cannot be generalized across all assessments. Associations among medical conditions, behavior, and functioning do not establish that treating a co-occurring problem changes an autism diagnosis.

SMART-NB remains an author-developed proposal. Its clinical profiles, demand formulations, seven-domain organization, rating anchors, weighting, missing-data rules, GFI, and visual interpretation have not been independently validated by the evidence reviewed here. The proposed definitions make testing possible but do not constitute content-validity or feasibility results. Existing frameworks already address integrated assessment; added value has not been demonstrated. The author's intellectual and potential commercial interests reinforce the need for independent evaluation.

## Conclusions

Assessment of children with autism is most clinically informative when diagnostic findings are connected with developmental context, medical and psychological needs, adaptive functioning, and everyday participation. Observational tools contribute to that process, while direct characterization of functioning helps identify needs that a diagnostic label or symptom score alone may not capture. The practical implication is to document how each relevant finding informs an individualized and revisable care priority.

SMART-NB proposes one explicit organization of that reasoning through complementary profiles, seven functional domains, individualized goals, and longitudinal review. Its contribution at this stage is a specification that can be critically examined and tested. The next step is independent operational refinement and feasibility work, followed by appropriate measurement studies and comparison with existing care. Any claim of clinical advantage should depend on demonstrated improvements in decisions, participation, or outcomes meaningful to children and families.
